# Frequency and Prognostic Relevance of *FLT3* Mutations in Saudi Acute Myeloid Leukemia Patients

**DOI:** 10.1155/2014/141360

**Published:** 2014-02-20

**Authors:** Ghaleb Elyamany, Mohammad Awad, Kamal Fadalla, Mohamed Albalawi, Mohammad Al Shahrani, Abdulaziz Al Abdulaaly

**Affiliations:** ^1^Department of Hematology and Blood Bank,Theodor Bilharz Research Institute, Giza 12411, Egypt, Egypt; ^2^Department of Central Military Laboratory, Prince Sultan Military Medical City, P.O. Box 7897, Riyadh 11159, Saudi Arabia; ^3^Department of Adult Clinical Hematology and Stem Cell Therapy, Prince Sultan Military Medical City, Riyadh, Saudi Arabia; ^4^Department of Pediatric Hematology/Oncology, Prince Sultan Military Medical City, P.O. Box 7897, Riyadh 11159, Saudi Arabia

## Abstract

The Fms-like tyrosine kinase-3 (*FLT3*) is a receptor tyrosine kinase that plays a key role in cell survival, proliferation, and differentiation of hematopoietic stem cells. Mutations of *FLT3* were first described in 1997 and account for the most frequent molecular mutations in acute myeloid leukemia (AML). AML patients with *FLT3* internal tandem duplication (ITD) mutations have poor cure rates the prognostic significance of point mutations; tyrosine kinase domain (TKD) is still unclear. We analyzed the frequency of *FLT3* mutations (ITD and D835) in patients with AML at diagnosis; no sufficient data currently exist regarding *FLT3* mutations in Saudi AML patients. This study was aimed at evaluating the frequency of *FLT3* mutations in patients with AML and its significance for prognosis. The frequency of *FLT3* mutations in our study (18.56%) was lower than many of the reported studies, *FLT3*-ITD mutations were observed in 14.4%, and *FLT3*-TKD in 4.1%, of 97 newly diagnosed AML patients (82 adult and 15 pediatric). Our data show significant increase of *FLT3* mutations in male more than female (13 male, 5 female). Our results support the view that *FLT3*-ITD mutation has strong prognostic factor in AML patients and is associated with high rate of relapse, and high leucocytes and blast count at diagnosis and relapse.

## 1. Introduction

*FLT3* (Fms-like tyrosine kinase-3), also known as FLK2 (fetal liver kinase-2) and STK1 (human stem cell kinase-1), was originally isolated as a hematopoietic progenitor cell-specific kinase and belongs to Class-III receptor tyrosine kinase (RTK) family to which c-Fms, c-Kit, and the PDGFR (platelet derived growth factor receptor) also belong [1]. Normal expression of *FLT3* is restricted to hematopoietic progenitor cells in the bone marrow (BM), thymus, and lymph nodes but is also found in other tissues such as placenta, brain, cerebellum, and gonads [2].

**Figure 1:**
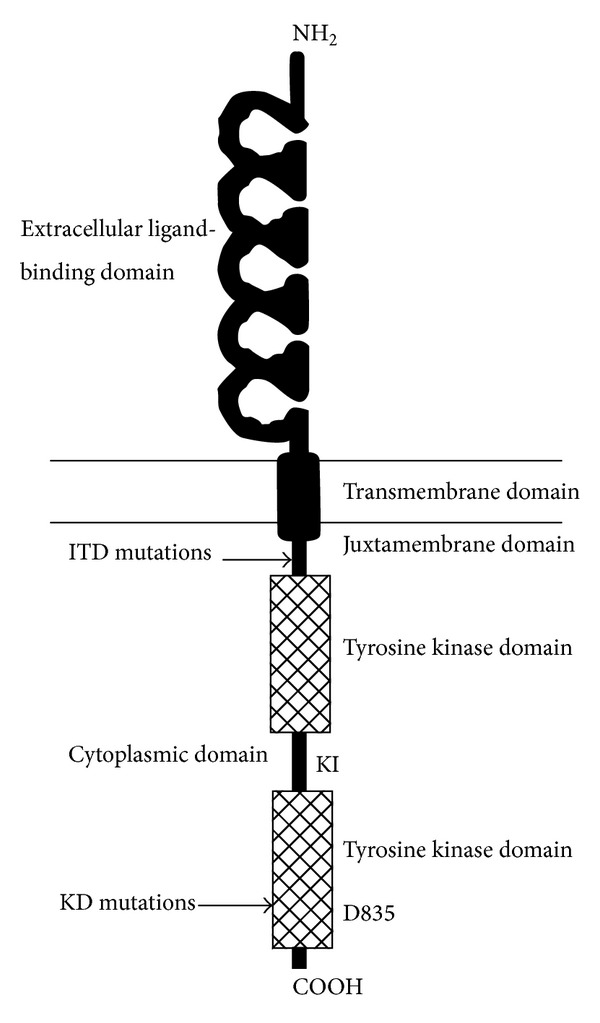
Diagram of *FLT3* structure. Shown in schematic fashion are the 5 immunoglobulin-like folds that make up the ligand-binding extracellular domain, single transmembrane domain, and cytoplasmic domain made up of a kinase domain interrupted by a kinase insert. The juxtamembrane domain where internal tandem duplications (ITDs) occur and aspartic acid 835 where most kinase domain mutations occur are indicated by arrows (Small D. *FLT3* Mutations: Biology and Treatment. Hematology Am Soc Hematol Educ Program. 2006).

*FLT3* plays a key role in cell survival, proliferation, and differentiation of hematopoietic stem cells [3]. The human *FLT3* gene is located on chromosome 13q12 and encompasses 24 exons. It encodes a membrane-bound glycosylated protein of 993 amino acids with a molecular weight of 158–160 kDa, as well as a nonglycosylated isoform of 130–143 kDa that is not associated with the plasma membrane [4]. The structure of *FLT3* is shown in Figure 1. *FLT3* is frequently overexpressed in acute leukemia.* FLT3* mutations occur in approximately 30% of acute myeloid leukemia (AML) patients and confer a poor prognosis [5].

The two major types of mutation that occur are internal tandem duplication (ITD) mutations of the juxtamembrane region and point mutations in tyrosine kinase domain (TKD), which frequently involve aspartic acid 835 of the kinase domain (D835). Both mutations result in constitutive activation of the receptor's tyrosine kinase activity in the absence of ligand [6].

Many studies have shown that AML patients with *FLT3*-ITD mutations have poor cure rates due to relapse. This has led to the development of a number of small molecule tyrosine kinase inhibitors (TKI) with activity against *FLT3* [7]. Adult patients usually have a higher prevalence of *FLT3*-ITD (24%) than pediatric AML patients. This observation may partially explain why adult AML has a poorer clinical outcome than pediatric AML [8]. Moreover, the clinical significance of *FLT3*-TKD mutation which is found in approximately 5–10% of AML patients is not clear yet, but several studies indicate that it is also an adverse prognostic indicator [8]. Clinically AML patients with *FLT3*-ITD tend to have higher WBC counts and an increased percentage of leukemic blasts [9]. *FLT3* mutations have also been seen in myelodysplastic syndrome (MDS) in about 3–5% of newly diagnosed patients and in MDS patients without *FLT3* mutations, they sometimes appear when these patients progress to AML [10].

This study which is considered the first study done in Saudi Arabia focuses on frequency and prognosis of the presence of* FLT3* mutations in adult and pediatric acute myeloid leukemia patients; only one study was done for detection of *FLT3* oncogene mutations in adult acute myeloid leukemia using conformation sensitive gel electrophoresis [11].

## 2. Materials and Methods

### 2.1. Patients

Bone marrow samples or blood samples from 97 patients with AML at diagnosis (66 BM and 31 blood samples) were screened for *FLT3* mutations. The range of age was one year old to 82 years (median 36 years, mean 37.8 years). Of the 97 patients, 94 (96.9%) had de novo AML, 1 (1%) had secondary AML after myelodysplastic syndrome (MDS) (s-AML), and 2 (2.1%) had AML after transformation from CML. The study was approved by the ethics and research committee of the institution.

Samples were evaluated by cytomorphology, multiparameter flow cytometry, cytogenetics, fluorescence in situ hybridization (FISH), and molecular genetics in parallel.

### 2.2. Methods

DNA was extracted using QIAamp DNA Kit (Qiagen) according to the manufacturer's instruction.

#### 2.2.1. Analysis of the ITD of the *FLT3* Gene

PCR reaction was composed of 200 ng of DNA, 50 mM KCL, 10 mM Tris-HCL, pH 8.3, 1.5 mM MgCL_2_, 0.001% (wt/vol) gelatin, 200 *μ*M dNTPs, 0.4 *μ*M of each published primer [29], and 1 U of gold *Taq* polymerase, in a volume of 50 *μ*L.

The PCR consisted of an initial incubation step at 95°C for 10 minutes followed by 35 cycles at 94°C for 30 seconds, 57°C for 60 seconds, and 72°C for 90 seconds and final extension step at 72°C for 10 minutes on a GeneAmp PCR system 9700 (Applied Biosystems). PCR products were analyzed on standard 3% agarose gels, and samples showing additional longer PCR products were considered *FLT3*/ITD+.

#### 2.2.2. Analysis of the D835 Mutation of the *FLT3* Gene

PCR reaction was set up as above using published primers [28]. PCR product was digested with EcoRV (Promega), at 37°C for 2 h. The digestion products were separated on a 3% agarose gel, and incomplete digestion indicated the presence of a mutant.

## 3. Statistical Analysis

The Kaplan-Meier technique was used to analyze the probability of overall survival (OS) and event-free survival (EFS). OS was calculated from time of diagnosis to death and EFS from time of diagnosis to death, evidence of persistent leukemia, or relapse. Continuous variables, such as white blood cell count and hemoglobin, were compared by using the Kruskal-Wallis test. Differences between means were considered as significant at *P* < 0.05.

## 4. Results

**Figure 2:**
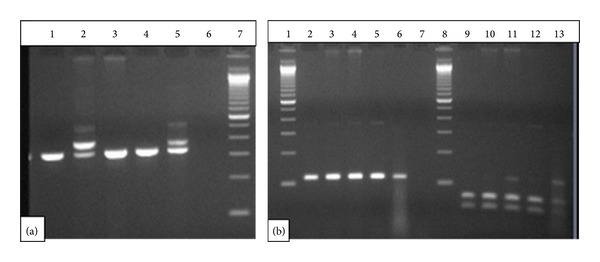
PCR analysis of *FLT3*-ITD and D835 mutations. (a) This gel shows patients positive for ITD lanes 2 and 5, patients negative for ITD lanes 1, 3, and 4, water control lane 6, and marker lane 7. (b) This gel shows undigested sample for D835 lanes 2–6, water control lane 7, marker lanes 1 and 8, digested sample lanes 9–13, positive patients lanes 11 and 13, and negative patients lanes 9, 10, and 12.

Table 1 summarizes the characteristics of the patients included in the study. Of the 97 AML patients studied, 47 were males (48.5%) and 50 were females (51.5%); 18 cases (13 males, 5 females) were positive for *FLT3* mutations with overall frequency of 18.55%. In these 18 *FLT3*-positive cases, 14/97 (14.43%) had *FLT3*-ITD and 4/97 (4.12%) were found to contain the D835 mutations (Figures 2(a) and 2(b)). None of the 3 secondary AML (MDS and CML) patients examined showed *FLT3*-ITD or D835 mutations. None of the patients had combination of *FLT3*-ITD and D835 mutation in the *FLT3* gene.

In *FLT3*-mutated patients median WBC was 65 × 10^9^/L compared to 12.5 × 10^9^/L in the rest of the patients. Peripheral blood blasts were elevated in *FLT3*-mutated group compared to *FLT3*-wild type (WT) patients (40% versus 8%, resp.).

Among *FLT3*-mutated patients, 8 cases died, 5 during induction chemotherapy and 3 during consolidation chemotherapy (1/8 D835+ and 7/8 ITD+), 7 cases relapsed within 4–20 months (1/7 D835 and 6/7 ITD+), and 3 cases (2/3 D835+ and 1/3 ITD+) achieved complete remission (CR).

As cytogenetic study was available only in a limited number of cases, correlation for cytogenetic analysis was not possible. In mutated group, cytogenetic and molecular studies revealed that 4 cases (22.2%) are associated with AML specific abnormalities, namely, PML-RARA/t (15; 17)(q22; q21), AML-ETO t(8; 21)(q22; q22), CBFB/inv(16)(p13; q22), and MLL (DC,BAR)/11q23. By conventional karyotype, it was not available for 5 cases because of a low number of analyzed metaphases; 3 cases were trisomy (2 cases with trisomy 8 and one case with trisomy 5) and 6 cases were with normal karyotype. Median overall survival was 10.0 months for *FLT3*-mutated patients and 20.0 months for WT patients (*P* = 0.031), and *FLT3*-positive patients had also a significantly shorter 2-year event-free survival (EFS) than *FLT3*/WT patients (*P* = 0.040) because of a higher relapse rate.

## 5. Discussion

*FLT3* mutations are one of the most frequent gene defects so far reported in AML, occurring in approximately 25–35% of patients [3, 28, 30–32]. *FLT3*-ITD represents one of the most frequent genetic alterations in AML. They show a frequency of 20% to 27% in AML in adults [22, 28] and of 10% to 16% in childhood cases [33, 34].

The overall frequency of *FLT3* mutations in our study (18.55%) is lower than most of the reported studies [3, 28, 30–32]; however, some reports agree with our report [21]. Also, the only study which was conducted in Saudi Arabia for *FLT3* mutations in AML patients showed frequency of 20.15% (26/129), close to our results [11]. The explanations of lower frequency of *FLT3* mutations in our study from other several studies may be explained by differences in the sizes of examined groups or might be due to population genetics, environmental factors, selected patient population studies, or because of differences of age as the median age of patients in this study was 36 years in comparison to comparative studies or a combination of the all.

Adult patients usually have a higher prevalence of *FLT-3*-ITD mutation than pediatric AML patients; this observation may partially explain why adult AML has a poorer clinical outcome than pediatric AML [8]. In our study, the frequency of *FLT3*-ITD mutation in adult AML was 14.43% which is lower than reported in other published studies [8, 19, 20, 22, 28, 35], but still in the variation range of frequency of ITD mutations (13–27%) reported by some study group [12, 21] (Table 2). In contrast to published studies the rate of *FLT3*-ITD mutation in pediatric AML (20%) is more than the adult and higher than most of the reported studies [33, 34, 36]. This difference in the rate most likely is attributed to small size of samples of pediatric AML (15 cases).

Similarly, the rate of *FLT3*-TKD mutation (D835) in our study is 4.12% (4/97) which is less frequent than *FLT3*-ITD mutation (14/97), these results are in accordance with the published studies on the frequency of *FLT3*-TKD in AML performed by many study groups [20, 22, 27, 28]; the rate of *FLT3*-TKD mutation (D835) in our study is almost very close to the largest study done by Bacher et al. [23] in which *FLT3*-TKD mutations were detected in 147 of 3082 (4.8%) patients. However, our results are slightly lower than many studies on the frequency of *FLT3*-TKD in AML (Table 3) which were performed by Abu-Duhier et al. [27], Yamamoto et al. [28], Thiede et al. [22], Moreno et al. [26], and Fröhling et al. [8]; according to these studies, *FLT3*-TKD mutation shows an incidence of 5.8% to 7.7% in AML. However, some of the reported studies show incidence higher than these studies ranged from 8 to 12% [22].

Our data are in accordance with those published by other groups [8, 13, 14, 17, 22, 29, 37–41] and showed that *FLT3*-ITD mutation has a strong prognostic factor in AML patients and associated disease progression with high rate of relapse and shorter overall survival; median overall survival was 10.0 months for *FLT3*-mutated patients and 20.0 months for WT patients (*P* = 0.031), and event-free survival (EFS) was also worse for *FLT3*-positive patients than *FLT3*-WT patients (*P* = 0.040**) **because of a higher relapse rate. Also high leucocytes count, high blast cells count in peripheral blood, resistant to therapy and confers a poor prognosis, Table 1. This has led to the development of a number of small molecule tyrosine kinase inhibitors (TKI) with activity against *FLT3* [7, 42]. Moreover, patients with low or absent levels of WT *FLT3*, consistent with homozygosity for the *FLT3*-ITD allele, appear to have a particularly dismal outcome [22].

Because of the low frequency, the prognostic significance of *FLT3*-TKD mutations is still unclear [23]. Many recent studies [21, 23, 43] showed that* FLT3*-TKD has little or no prognostic significance in AML patients. In this study, according to our data unlike the *FLT3*-ITD mutation, which are associated with inferior survival, prognosis was not influenced by mutation of *FLT3*-TKD. Moreover, Bacher et al. [23] found an additional favorable impact of *FLT3*-TKD on *EFS* in prognostically favorable AML with NPM1 or CEBPA mutations. However, Yamamoto et al. [28] revealed that, in contrast to our study, D835 mutations were not significantly related to the leukocytosis but tended to worsen disease-free survival.

As a surprising finding in our study, the rate of *FLT3* mutations in male (13/18; 72.2%) is higher than in female (5/18; 27.8%); this finding may be the first one reported in English literature showing the significant difference between male and female (*P* = 0.025). One small study was done on 30 AML patients with frequency of *FLT3*/ITD mutation in AML which was 4/30 (13.3%); three were males and one female [12]. However, these results should be treated with reservation, due to the relatively small sample sizes. These differences in male/female ratio in our study from several reported studies may be due to population genetics, environmental factors, relative small sample sizes, or a combination of all and further research and investigation are required.

As one interesting rare finding, one adult AML patient in our cohort study who did not have *FLT3* mutations at diagnosis has been found to acquire *FLT3*/ITD at the time of relapse; this finding was reported by another study group [10] and raises the importance of assessing *FLT3* gene on all relapses. This finding also suggests that* FLT3* mutations are unstable and that there is potential clinical value in continuously monitoring *FLT3* mutation status [44].

## 6. Conclusion

Currently no sufficient data exist regarding *FLT3* mutations in Saudi patients. This study was aimed at evaluating the prevalence of *FLT3* mutations in patients with AML and its significance for prognosis. The frequency of *FLT3* mutations in our study was lower than (18.56%) other reported studies; however, some reports agree with our report or are very close. Our data as other several reports support the view that *FLT3*/ITD has strong prognostic factor in AML patients and is associated with high rate of relapse, is resistant to therapy, and confers a poor prognosis. Our data show increase of *FLT3* mutations in male more than female which need more research studies using large size samples. This study also raises the importance of assessing* FLT3* gene on all relapses as one case which did not have *FLT3* mutations at diagnosis has been found to acquire *FLT3*/ITD at the time of relapse. Early detection of *FLT3* mutations and an intensification of induction therapy might thus be useful for this group of patients to overcome the poor prognosis.

## Figures and Tables

**Table 1:** Summary of the characteristics of the patients included in the study.

Parameter	*FLT3*-mutated patients (*n* = 18)	*FLT3* WT patients (*n* = 79)
Male : female	13 : 5	34 : 45
Median age (years)	38	35
Median WBCs count	65 × 10^9^/L	12.5 × 10^9^/L
Median platelets count	53 × 10^9^/L	71 × 10^9^/L
Median hemoglobin	9.1 g/dL	10 g/dL
Median PB blasts	40%	8%
History of AML		
De novo AML	18/94	76/94
Secondary s-AML	0/3	3/3
*FLT3* mutation rate and OS		
*FLT3*-ITD	14/97 (14.43%)	NA
*FLT3*-D835	4/97 (4.12%)	NA
Total	**18/97 (18.55%)**	NA
Median OS (months)	10	20
Cytogeneticanalysis		
Available for FISH	18	56
Available for karyotype	13	40
t (8; 21)	1	6
t (15; 17)	1	3
inv 16/t (16; 16)	1	3
11q23/MLL	1	2
Non recurrent translocations	0	5
+8	2	5
+5	1	1
+13	0	2
+21	0	1
−8	0	1
5q−/−5	0	2
7q−/−7	0	2
Complex karyotype	0	2
Hyperdiploid	0	2
Other aberrations	0	4

WT: wild type; OS: overall survival; FISH: fluorescence in situ hybridization.

**Table 2:** Frequency of *FLT3*/ITD mutations in the current study and in previous studies.

Reference (year)	Total, *n*	FLT-ITD+ %
Our study (2013)	97	14.4
Ishfaq et al. [12] (2012)	30	13.3
Xu et al. [13] (2012)	216	20.8
Ding et al. [14] (2012)	656	27.1
Zaker et al. [15] (2010)	212	18.0
Wang et al. [16] (2010)	76	19.7
Al-Tonbary et al. [17] (2009)	30	20.0
Gari et al. [11] (2008)	129	11.6
Suzuki et al. [18] (2007)	60	20.0
Wang et al. [19] (2005)	143	25.9
Auewarakul et al. [20] (2005)	256	27.3
Sheikhha et al. [21] (2003)	80	10.0
Fröhling et al. [8] (2002)	224	32.0
Thiede et al. [22] (2002)	979	20.4

**Table 3:** Frequency of *FLT3*/TKD mutations in the current study and in previous studies.

Reference (year)	Total, *n*	FLT-TKD %
Our Study (2013)	97	4.1
Ding et al. [14] (2012)	656	7.0
Zaker et al. [15] (2010)	212	6.0
Gari et al. [11] (2008)	129	8.5
Bacher et al. [23] (2008)	3082	4.8
Mead et al. [24] (2007)	1107	11.5
Auewarakul et al. [20] (2005)	256	5.9
Wang et al. [19] (2005)	143	6.3
Andersson et al. [25] (2004)	109 (<60 y)	10.1
Moreno et al. [26] (2003)	208	9.6
Sheikhha et al. [21] (2003)	80	7.5
Thiede et al. [22] (2002)	979	7.7
Fröhling et al. [8] (2002)	224	14.0
Abu-Duhier et al. [27] (2001)	97	7.2
Yamamoto et al. [28] (2001)	429	7.0

## Abbreviations

*FLT3*: Fms-like tyrosine kinase-3
FLK2: Fetal liver kinase-2
STK1: Human stem cell kinase-1
BM: Bone marrow
AML: Acute myeloid leukemia
ITD: Internal tandem duplication
TKD: Tyrosine kinase domain
RTK: Receptor tyrosine kinase
PDGFR: Platelet derived growth factor receptor
MDS: Myelodysplastic syndrome
WT: Wild type
OS: Overall survival
FISH: Fluorescence in situ hybridization
EFS: Event-free survival
TKI: Tyrosine kinase inhibitors.
